# The mitogenome of the jumping bristletail *Trigoniophthalmus alternatus* (Insecta, Microcoryphia) and the phylogeny of insect early-divergent lineages

**DOI:** 10.1080/23802359.2019.1660592

**Published:** 2019-09-03

**Authors:** Chiara Leo, Francesco Nardi, Francesco Frati, Pietro Paolo Fanciulli, Claudio Cucini, Matteo Vitale, Claudia Brunetti, Antonio Carapelli

**Affiliations:** Department of Life Sciences, University of Siena, Siena, Italy

**Keywords:** Basal hexapods, Ectognatha, Microcoryphia, Diplura, Zygentoma, Mitogenomics, Phylogeny

## Abstract

The complete mitochondrial genome of the machilid *Trigoniophthalmus alternatus* (Silvestri 1904) is herein described and applied to phylogenetic analyses, inclusive of the most early-divergent lineages of hexapods. Both gene content and order generally conform with the organization of the arthropods’ mitochondrial genome. One gene translocation involving *trnA* is the autapomorphic character observed in this species. Another peculiar molecular feature is the long size of the A + T-rich region, due to the occurrence of repeat units. The phylogenetic analyses support the typical placement, along the hexapods’ tree, of Ectognatha, Monocondylia and Dicondylia, with Diplura as the adelphotaxon of all true insects.

Total DNA of one specimen of *Trigoniophtalmus alternatus* (Voucher specimen ID: TAL_05, preserved at Life Sciences Department of University of Siena; Collection site: Siena, Italy, 43°24′7.81″N 11°21′52.62″E) was isolated, amplified, and sequenced as described in Carapelli et al. (2007).

The complete mtDNA of *T. alternatus* was assembled using the software Sequencher 4.4.2 (Gene Codes Corporation, Ann Arbor, MI, USA). The consensus sequence was submitted to the tRNAs secondary structure prediction online tool ARWEN (Laslett and Canbäck 2008). Accordingly, the 22 tRNA-encoding sequences were then mapped on the genome contig. Boundaries of protein-encoding genes were also detected searching for their start and stop codons. The rDNA genes were identified drawing their putative secondary structure at the 5′- and 3′- ends of both *rrnS* and *rrnL*. The annotated genome sequence was deposited in GenBank under the accession number: NC010532.

The mitogenome of *T. alternatus* contains the common set of 37 genes, plus a non-coding DNA fragment (A + T-rich region) where the sequences that control both replication and transcription of the mtDNA are found. This complete genome sequence is the longest (16,197 bp in length) mtDNA among all Microcoryphia species so far investigated. In this taxon, the nucleotide content of the J-stand is biased toward A and T nucleotides (A = 38.8%, T = 32.6%, and G = 10.1%). All PCGs start with canonical Methionine codons (either ATA or ATG) except *cox1* (that starts with TTG, that encodes for Leucine); complete TAA stop codons are found in all genes apart from *cox1* and *nad4L* (both ending with truncated TA–).

Within the long A + T-rich region (1150 bp in size), there are three almost identical direct repeats of 217 bp (plus an additional partial repeat unit), as previously observed in other mtDNAs (Nardi et al. 2012).

The gene order of Microcoryphia is similar to that ancestral for Pancrustacea (Boore et al. 1998) with the exception of the tRNA cluster A-R-N-S-E-F, that is rearranged to: A-R-N-E-F in *Nesomachilis australica*, A-R-N-S-E-Y-F in *Petrobius brevistylis* and R-N-S-E-A-F in *T. alternatus*. No shared gene rearrangement among Microcoryphia species is observed.

The *T. alternatus* mtDNA genes were aligned with those of other 28 species (Figure 1), concatenated and used for phylogenetic analyses. The final alignment, obtained with RevTrans 1.4 (Wernersson and Pedersen 2003), was deprived of positions of dubious homology with the online tool GBlock (Castresana 2000). The resulting nucleotide matrix was partitioned into 39 charsets (one for each of the three codon positions of the 13 PCGs) and tested with the software PartitionFinder 2.1.1 (GTR + I and GTR + I + Γ models were selected; Lanfear et al. 2016). Two independent runs (all nucleotides and first and second codon positions only) were processed with the software MrBayes 3.2 (Ronquist et al. 2012), with four chains for 10^6^ generations and sampling one tree/1000 iterations (with 25% of burn-in). The resulting trees both support the monophyly of Ectognatha, Monocondylia, Dicondylia, Pterygota, Diplura, Ephemeroptera, Microcoryphia and Odonata (Figure 1). Zygentoma is the only high-ranking taxon that results paraphyletic (Figure 1).

**Figure 1.**
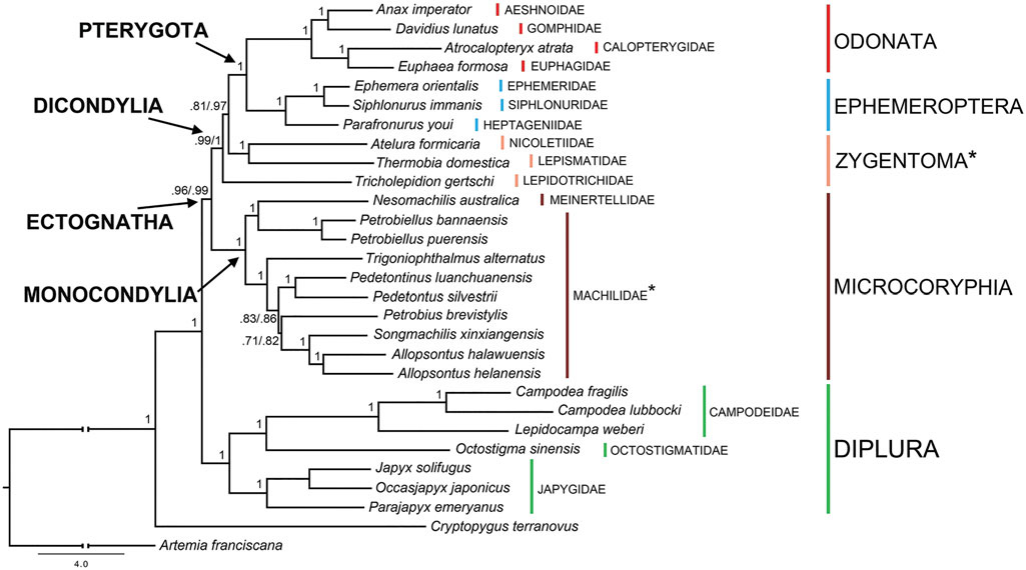
Bayesian phylogeny based on the concatenated set of 13 mitochondrial protein-encoding genes of the following basal hexapod lineages used in this study: *Campodea fragilis* DQ529236, *Campodea lubbocki* DQ529237 and *Lepidocampa weberi* JN990601 (Campodeidae; Diplura); *Octostigma sinensis* JN990598 (Octostigmatidae; Diplura); *Japyx solifugus* NC007214, *Occasjapyx japonicus* JN990600 and *Parajapyx emeryanus* JN990599 (Japygidae; Diplura); *Allopsontus helanensis* KJ754501, *Allopsontus halawuensis* KJ754500, *Nesomachilis australica* NC006895, *Pedetontinus luanchuanensis* KJ754502, *Pedetontus silvestrii* EU621793, *Petrobiellus bannaensis* KJ754503, *Petrobiellus puerensis* KJ754504, *Petrobius brevistylis* NC007688, *Songmachilis xinxiangensis* NC021384 and *Trigoniophthalmus alternatus* EU016193 (Microcoryphia); *Atelura formicaria* EU084035, *Thermobia domestica* AY639935 and *Tricholepidion gertschi* AY191994 (Zygentoma); *Ephemera orientalis* NC012645, *Parafronurus youi* NC011359 and *Siphlonurus immanis* NC013822 (Ephemeroptera); *Atracalopteryx atrata* NC027181 and *Eupohaea formosa* NC014493 (Zygoptera; Odonata); *Anax imperator* NC031821 and *Davidius lunatus* NC012644 (Anisoptera; Odonata). The collembolan *Cryptopygus terranovus* NC037610 and the crustacean *Artemia franciscana* NC001620 have been used as outgroups. Double branch-support on nodes (i.e. when different among alternative data sets) refers to analyses based on complete set of nucleotide codon positions/first and second only, respectively. *refers to paraphyletic groups.
